# Supplementary material for: The role of connectivity on COVID-19 preventive approaches
Source: PLoS One. 2022 Sep 1;17(9):e0273906. doi: 10.1371/journal.pone.0273906 (PMC9436065; doi:10.1371/journal.pone.0273906)
Supplement: S1 Fig — Top panel: An Erdős-Rényi graph (left hand side) and a power-law degrees graph (right hand side). For an easy visualization, the parameters where set to N = 200, μe = 12, = 3. Bottom panel: Degree distribution of the power law graph in log- log scale. (DOCX) [file pone.0273906.s001.docx]

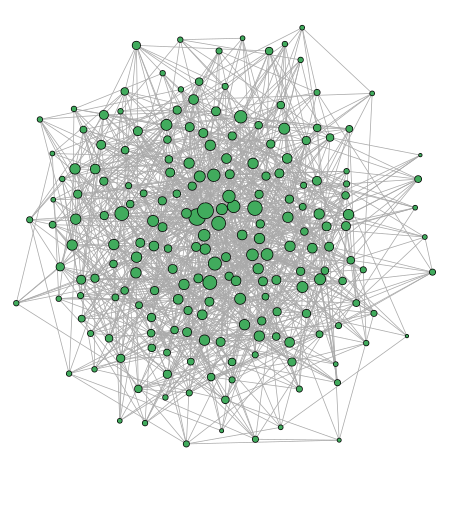

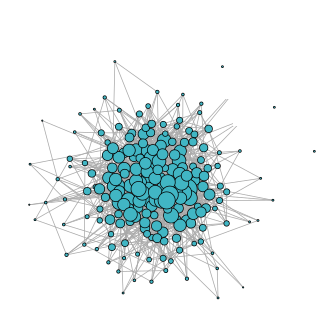


**
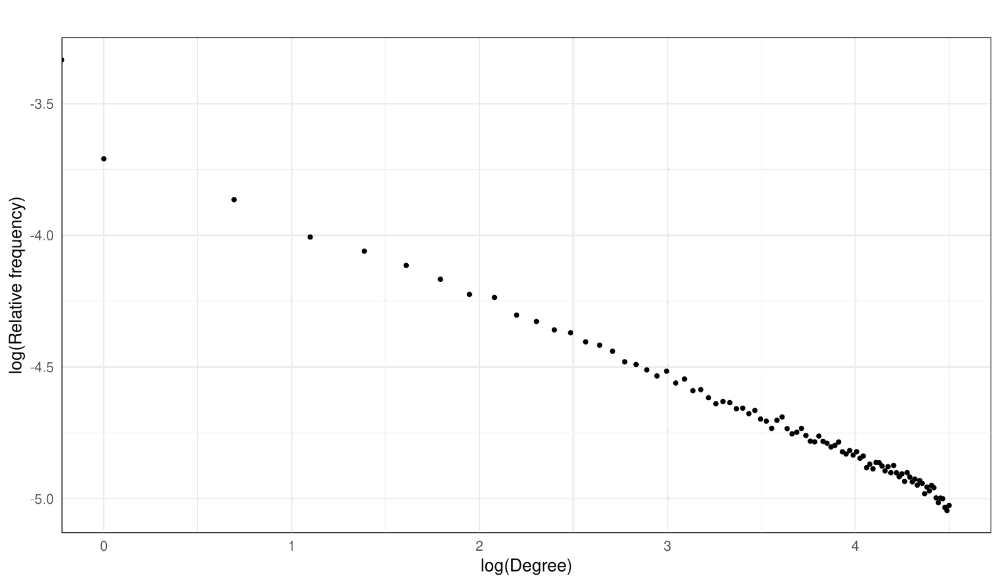
**

**S1 Fig.** Top panel: An Erdős-Rényi graph (left hand side) and a power-law degrees graph (right hand side). For an easy visualization, the parameters where set to N = 200, μe = 12,  =3. Bottom panel : degree distribution of the power law graph in log- log scale.
