# Supplementary material for: The role of connectivity on COVID-19 preventive approaches
Source: PLoS One. 2022 Sep 1;17(9):e0273906. doi: 10.1371/journal.pone.0273906 (PMC9436065; doi:10.1371/journal.pone.0273906)
Supplement: S2 Fig — Susceptible individuals can become infected with probability Pi if they have an infected neighbor. Infected individuals remain infected for an exponential random time with mean Tr. At the end of this infectious period they can recover with probability 1-Pd or die with probability Pd. (DOCX) [file pone.0273906.s002.docx]

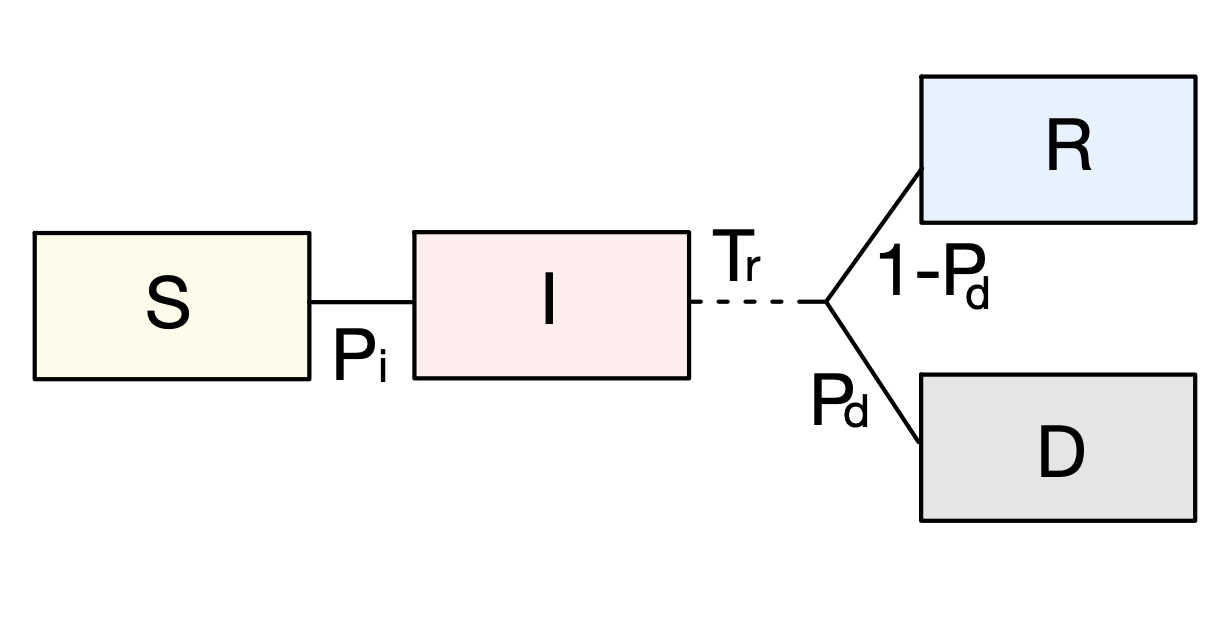


**S2 Fig.** Transition rates of our SIRD model. Susceptible individuals can become infected with probability Pi if they have an infected neighbor. Infected individuals remain infected for an exponential random time with mean Tr. At the end of this infectious period they can recover with probability 1-Pd or die with probability Pd .
