# Supplementary material for: The role of connectivity on COVID-19 preventive approaches
Source: PLoS One. 2022 Sep 1;17(9):e0273906. doi: 10.1371/journal.pone.0273906 (PMC9436065; doi:10.1371/journal.pone.0273906)
Supplement: S3 Fig — The curves represent the proportion of individuals in each category as functions of time. Each curve corresponds to the mean over 30 repetitions. A, C: Erdős-Rényi graphs, B, D: Power-law degree, A, B: e = 5. C, D: e = 10. The rest of the parameters are pi = 0.5 and = 3. (DOCX) [file pone.0273906.s003.docx]

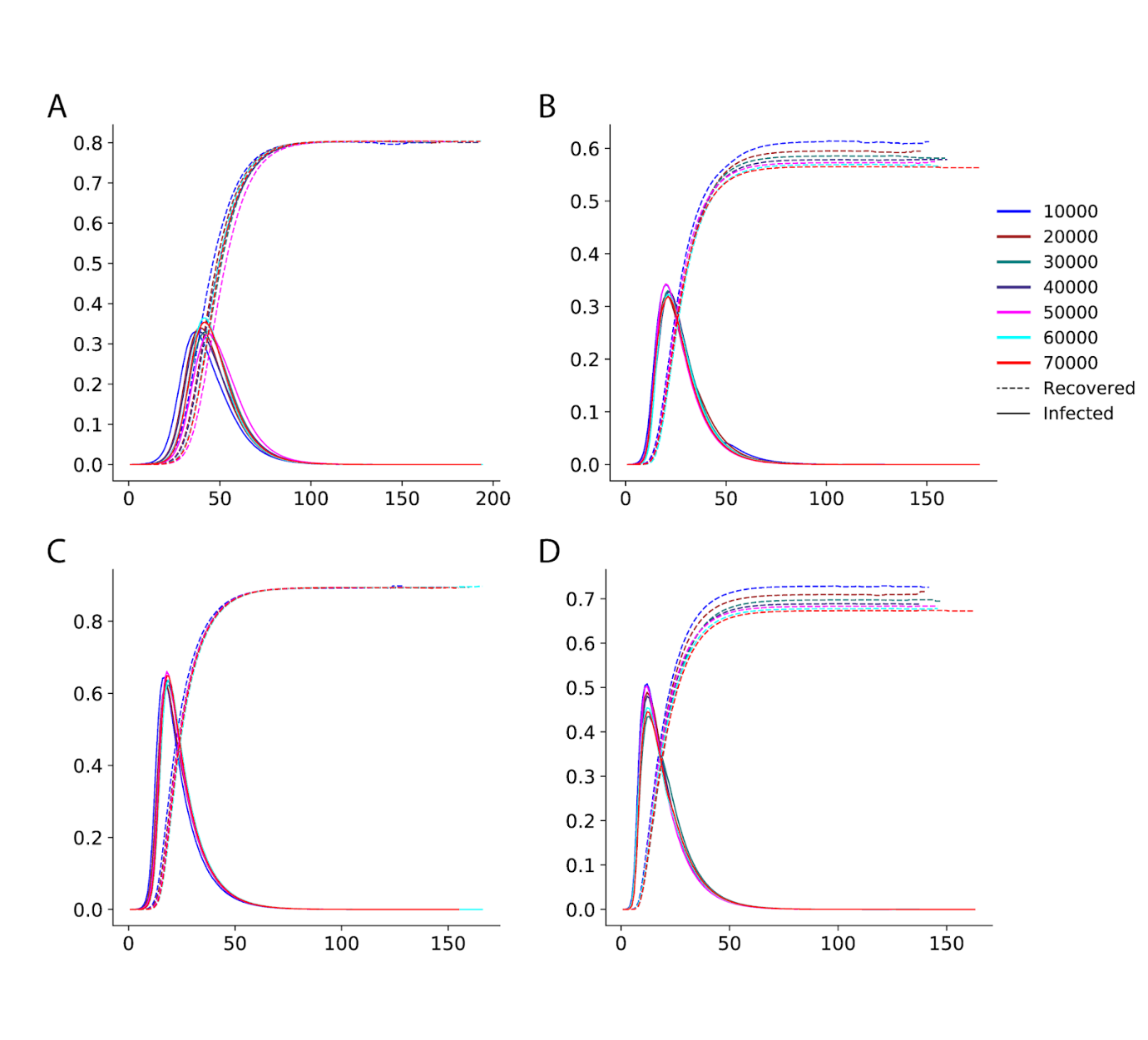


**S3 Fig.** Infected and recovered curves for population sizes 10 000, 20 000, 30 000, 40 000, 50 000, 60 000 and 70 000. The curves represent the proportion of individuals in each category as functions of time. Each curve corresponds to the mean over 30 repetitions.  A, C: Erdős-Rényi graphs, B, D: Power-law degree, A, B: e = 5. C, D: e = 10. The rest of the parameters are pi= 0.5 and =3.
