# Supplementary material for: The role of connectivity on COVID-19 preventive approaches
Source: PLoS One. 2022 Sep 1;17(9):e0273906. doi: 10.1371/journal.pone.0273906 (PMC9436065; doi:10.1371/journal.pone.0273906)
Supplement: S4 Fig — Top panels: Proportion of infected individuals through time (color enveloppes indicate the standard deviation computed across 30 replicates). Bottom panels: Distribution of the total number of infected individuals for 30 different simulations. The lockdown starts when the cumulative number of infected individuals is 10%. In the top panels, the dashed lines show the beginning and the end of the lockdowns (and its standard deviation indicated by a horizontal line). In the bottom panel dashed lines correspond to the average proportion of infected individuals for each condition. (DOCX) [file pone.0273906.s004.docx]

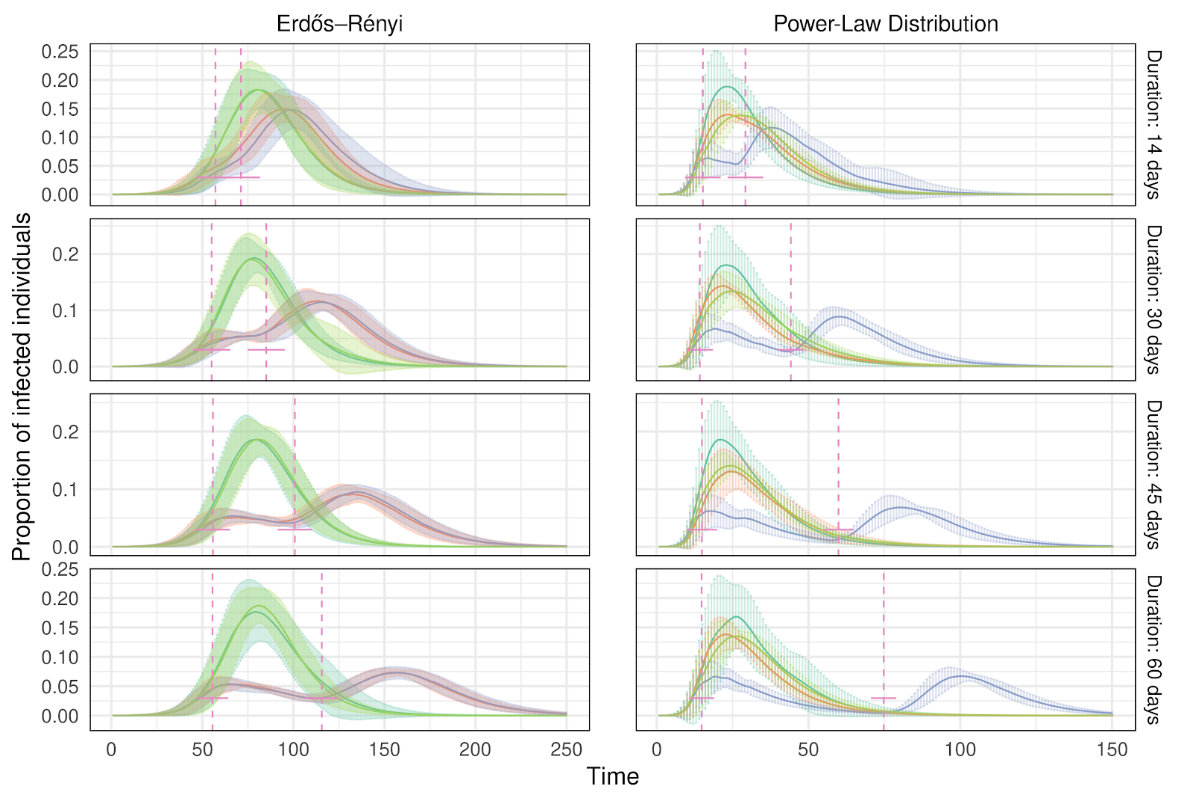


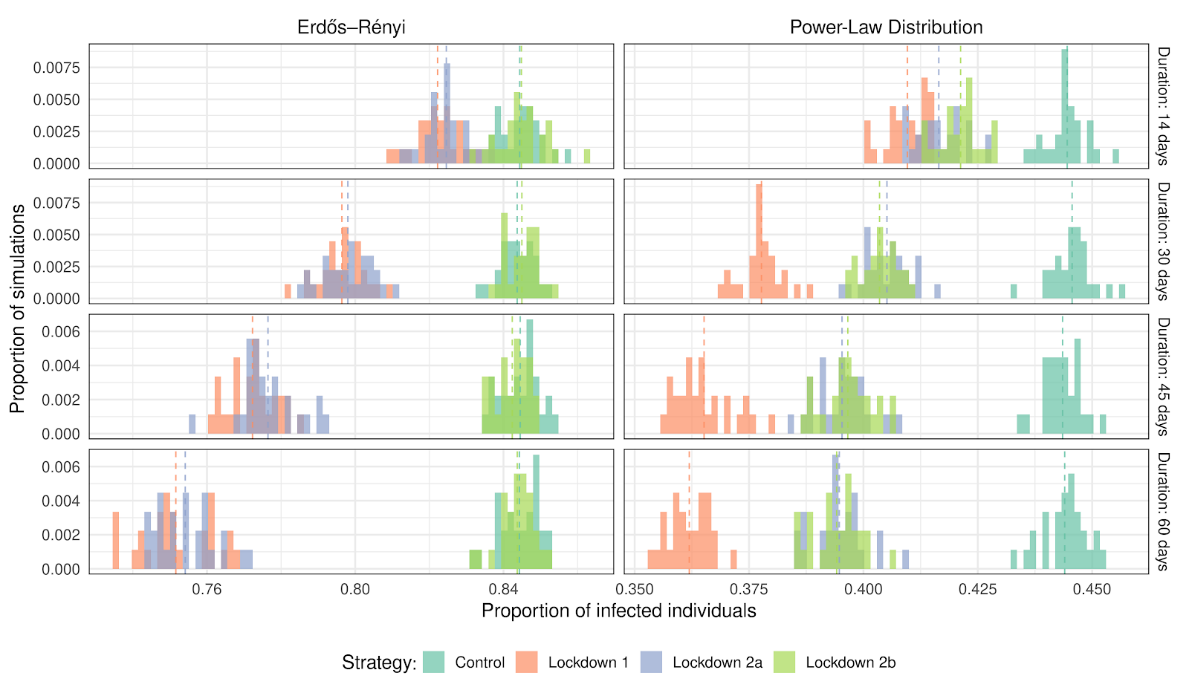


**S4 Fig.** Effect of the duration of the lockdown for different strategies. Top panels: proportion of infected individuals through time (color enveloppes indicate the standard deviation computed across 30 replicates). Bottom panels: distribution of the total number of infected individuals for 30 different simulations. The lockdown starts when the cumulative number of infected individuals is 10%.  In the top panels, the dashed lines show the beginning and the end of the lockdowns (and its standard deviation indicated by a horizontal line). In the bottom panel dashed lines correspond to the average proportion of infected individuals for each condition.
