# Supplementary material for: The role of connectivity on COVID-19 preventive approaches
Source: PLoS One. 2022 Sep 1;17(9):e0273906. doi: 10.1371/journal.pone.0273906 (PMC9436065; doi:10.1371/journal.pone.0273906)
Supplement: S5 Fig — The duration is fixed as 30 days, and we vary the cumulative proportion of infected individuals at the start of the lockdown tL. Top panels: Proportion of infected individuals through time (color enveloppes indicate the standard deviation computed across 30 replicates). Bottom panels: Distribution of the total number of infected individuals for 30 different simulations. The dashed lines have the same meaning as in S3 Fig. Observe that for tL = 1%, lockdowns have no substantial effect on the maximum of the infection curve in the Erdős-Rényi case, and the same holds for lockdown strategy 2a in the power-law degree case. A lockdown started later (at tL = 5% or 10%) is better with this respect. (DOCX) [file pone.0273906.s005.docx]

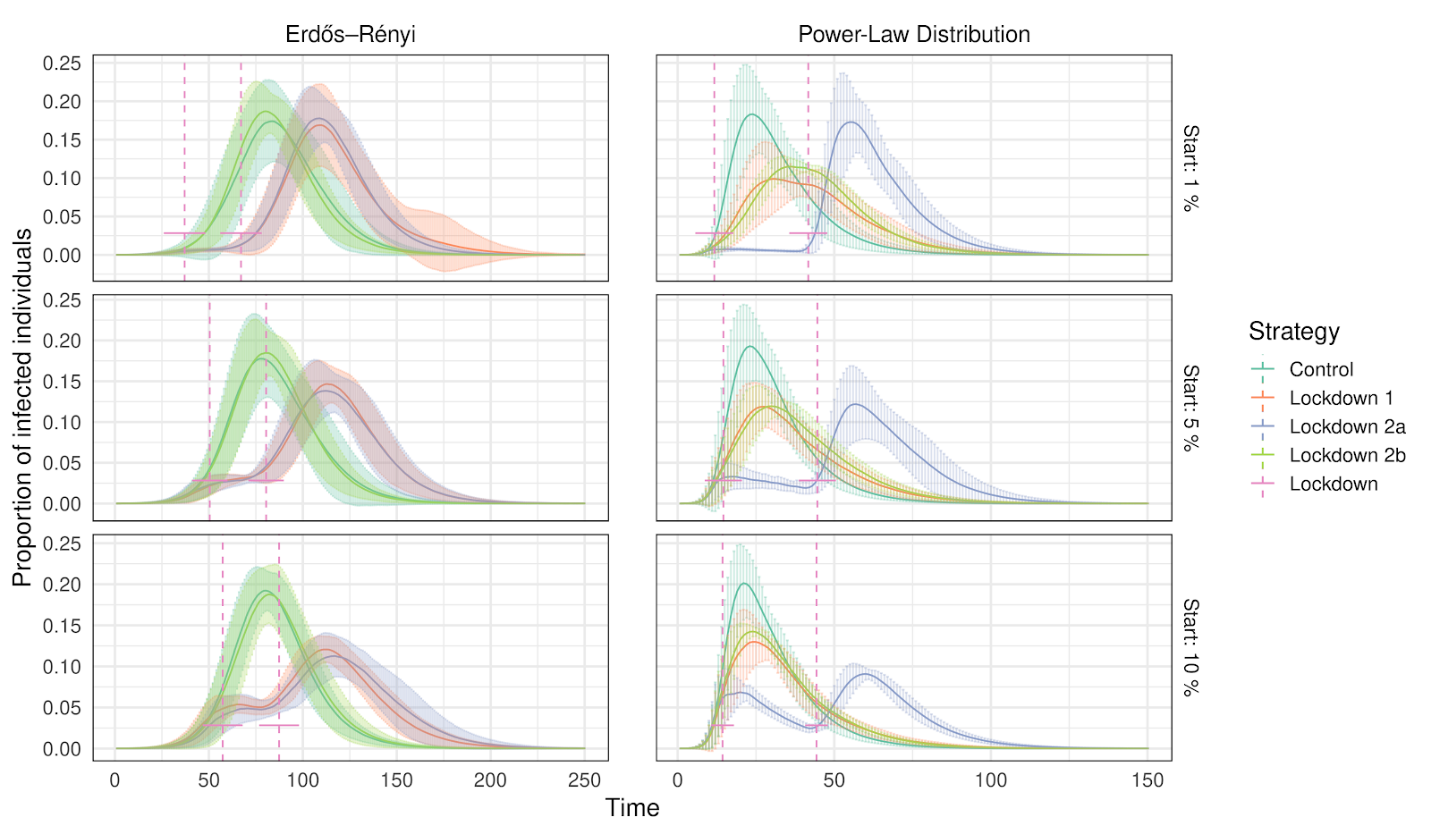


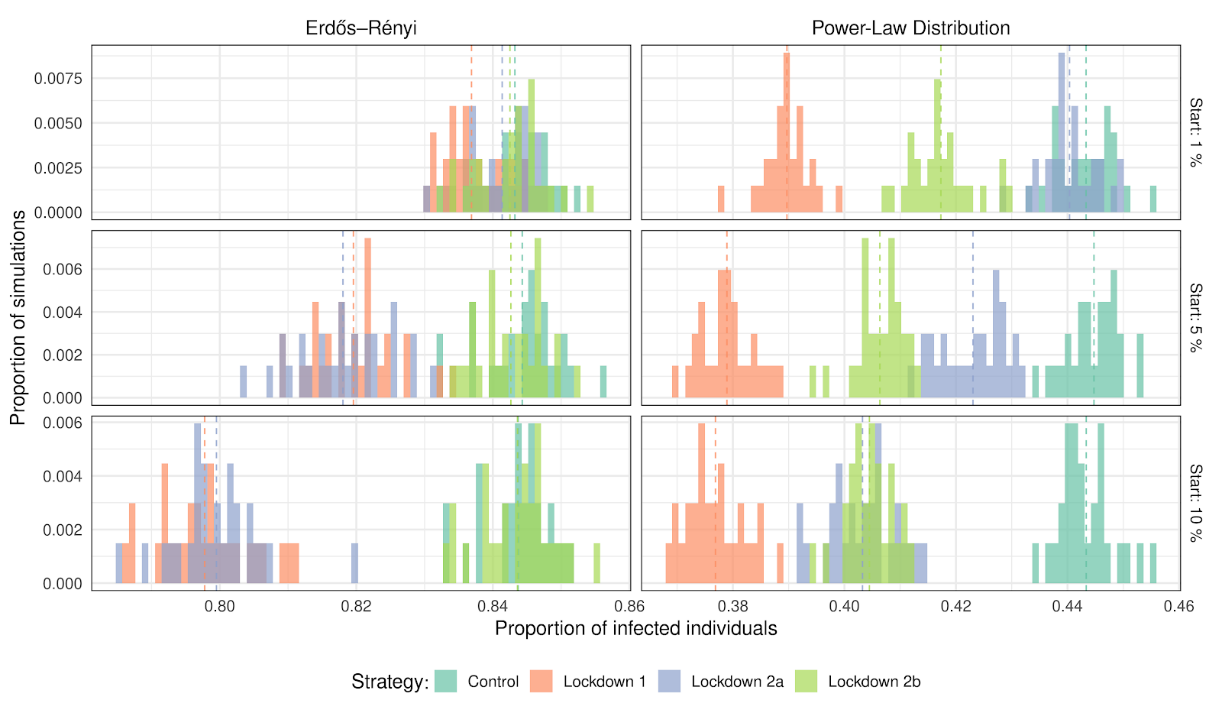


**S5 Fig.** Effect of the starting time for the lockdown. The duration is fixed as 30 days, and we vary the cumulative proportion of infected individuals at the start of the lockdown tL. Top panels: proportion of infected individuals through time (color enveloppes indicate the standard deviation computed across 30 replicates). Bottom panels: distribution of the total number of infected individuals for 30 different simulations. The dashed lines have the same meaning as in S3 Fig. Observe that for tL=1%, lockdowns have no substantial effect on the maximum of the infection curve in the Erdős-Rényi case, and the same holds for lockdown strategy 2a in the power-law degree case. A lockdown started later (at tL=5% or 10%) is better with this respect.
