# Supplementary material for: The role of connectivity on COVID-19 preventive approaches
Source: PLoS One. 2022 Sep 1;17(9):e0273906. doi: 10.1371/journal.pone.0273906 (PMC9436065; doi:10.1371/journal.pone.0273906)
Supplement: S6 Fig — The first panel, labeled “control” represents the degree distribution of the contact network before any intervention (DOCX) [file pone.0273906.s006.docx]

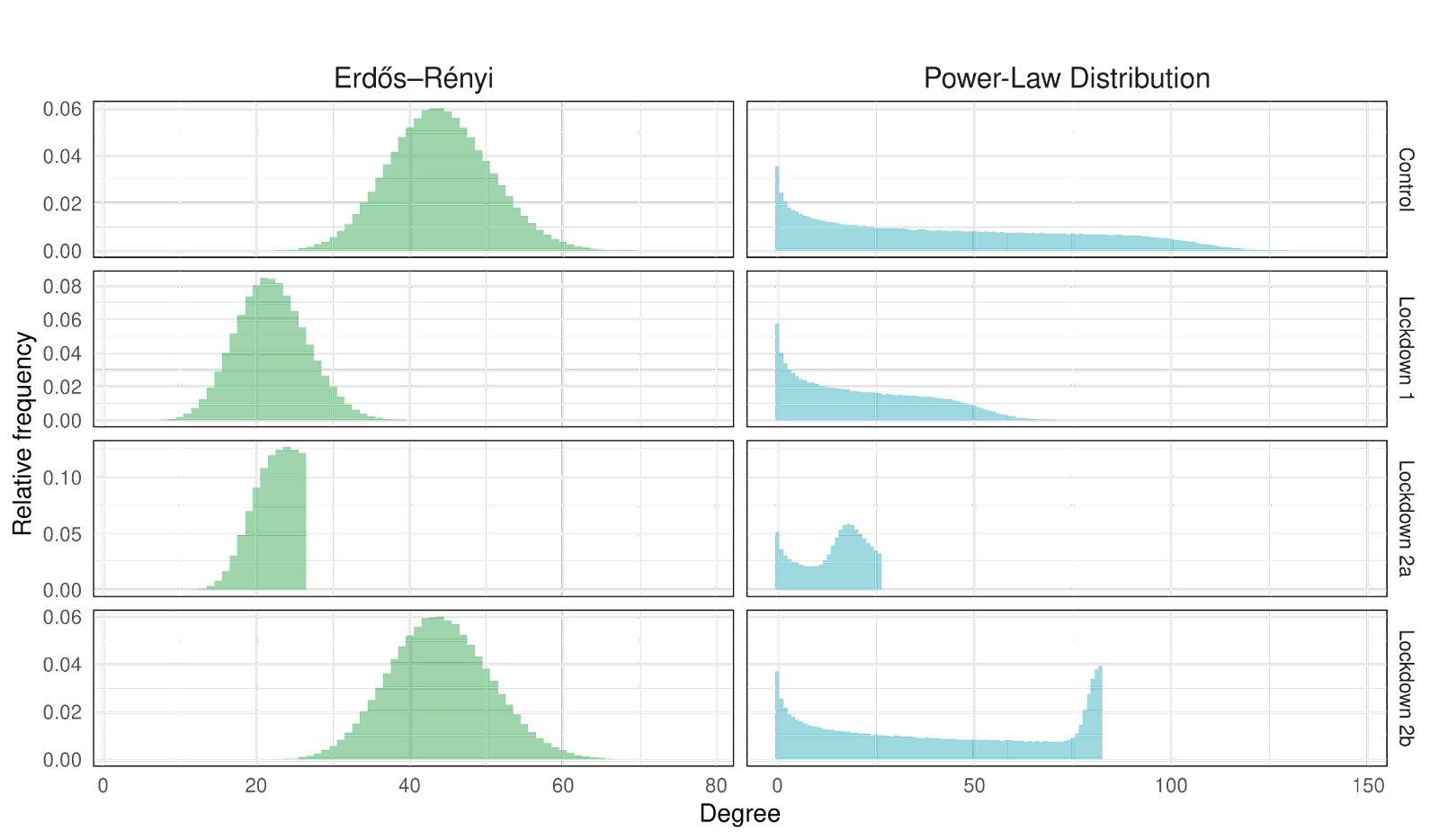


**S6 Fig.** Distribution of the number of risky interactions (edges) for the Erdős-Rényi and Power Law networks, for the different lockdown strategies. The first panel, labeled “control” represents the degree distribution of the contact network before any intervention
