# Supplementary material for: The role of connectivity on COVID-19 preventive approaches
Source: PLoS One. 2022 Sep 1;17(9):e0273906. doi: 10.1371/journal.pone.0273906 (PMC9436065; doi:10.1371/journal.pone.0273906)
Supplement: S7 Fig — The plot shows the proportion of infected at the end of the infection for 30 repetitions. The number of doses of the vaccine represents 25% of the population size (N = 20000). Different starting times are shown in the different panels (when 0, 10 and 30% of the individuals have been infected). On the top right panel, when vaccinating the most connected, the epidemic always died out quickly, before infecting at least 50 individuals, which is the minimum required to be considered a successful simulation (see Methods). (DOCX) [file pone.0273906.s007.docx]

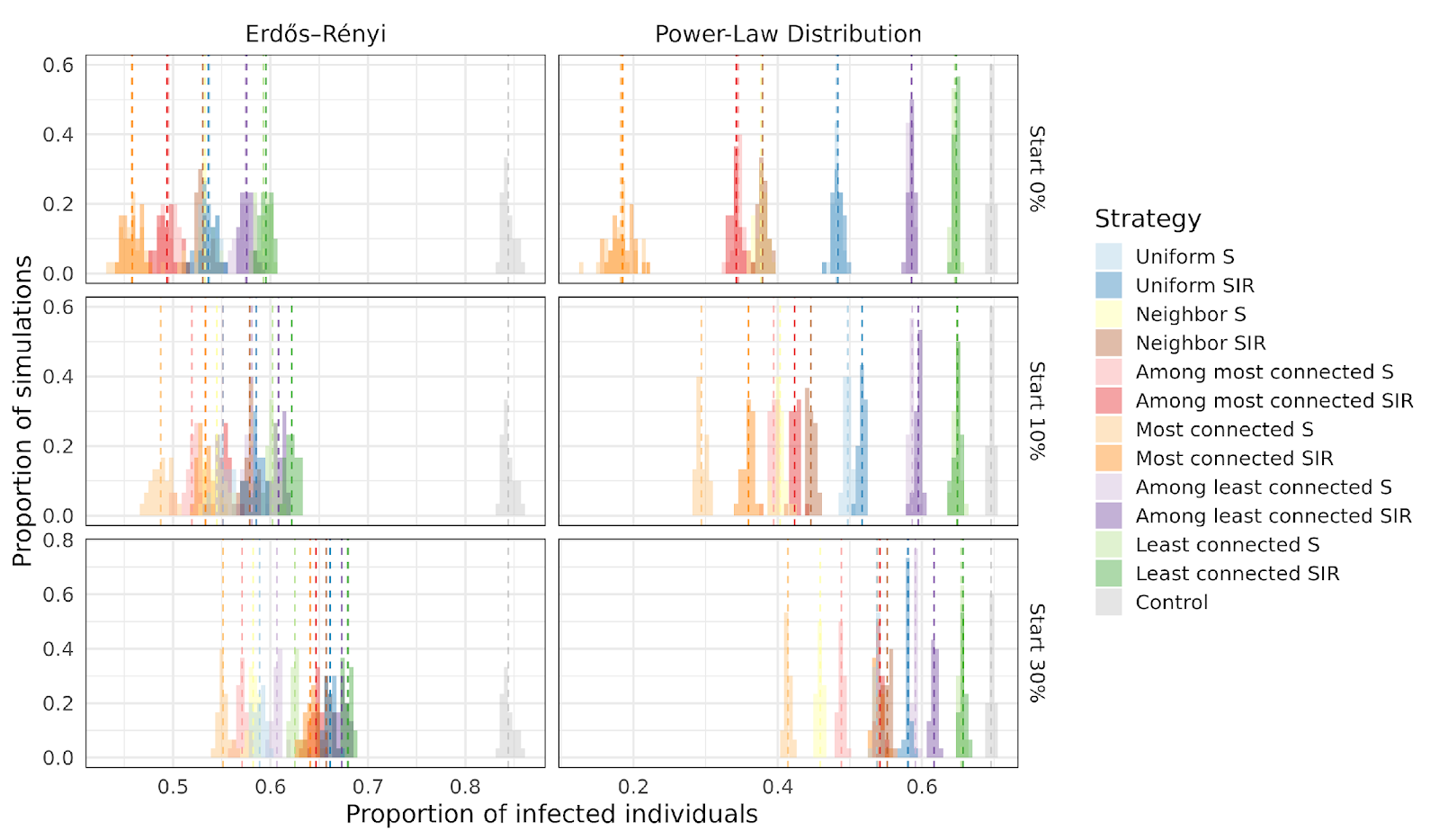


**S7 Fig.** Proportion of infected and dead individuals for all the vaccination strategies. The plot shows the proportion of infected at the end of the infection for 30 repetitions. The number of doses of the vaccine represents 25% of the population size (N = 20000).  Different starting times are shown in the different panels (when 0, 10 and 30% of the individuals have been infected). On the top right panel, when vaccinating the most connected, the epidemic always died out quickly, before infecting at least 50 individuals, which is the minimum required to be considered a successful simulation (see Methods).
