# Supplementary material for: The role of connectivity on COVID-19 preventive approaches
Source: PLoS One. 2022 Sep 1;17(9):e0273906. doi: 10.1371/journal.pone.0273906 (PMC9436065; doi:10.1371/journal.pone.0273906)
Supplement: S8 Fig — Plots show the total number of infected individuals in 30 simulations for the Erdős-Rényi (right) and power-law (left) graphs. From top to bottom we increase the number of individuals that we can vaccinate (10, 25, 50%). The time of vaccination is when the cumulative number of infected reaches 30% of the populations (tV = 30%). (DOCX) [file pone.0273906.s008.docx]

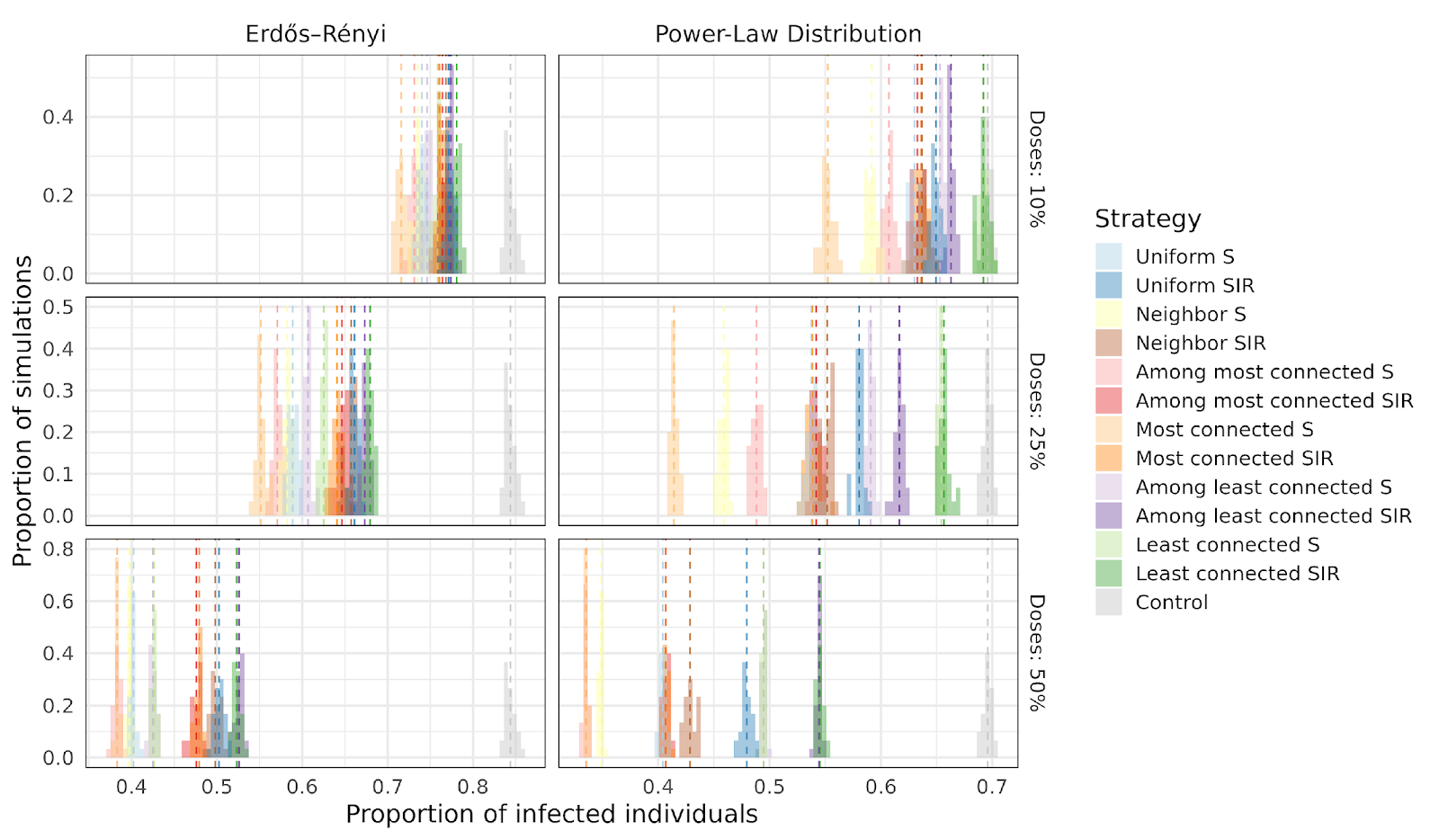


**S8 Fig.** Effect of the number of doses of the vaccine. Plots show the total number of infected individuals in 30 simulations for the Erdős-Rényi (right) and power-law (left) graphs. From top to bottom we increase the number of individuals that we can vaccinate (10, 25, 50%). The time of vaccination is when the cumulative number of infected reaches 30% of the populations (tV= 30%).
